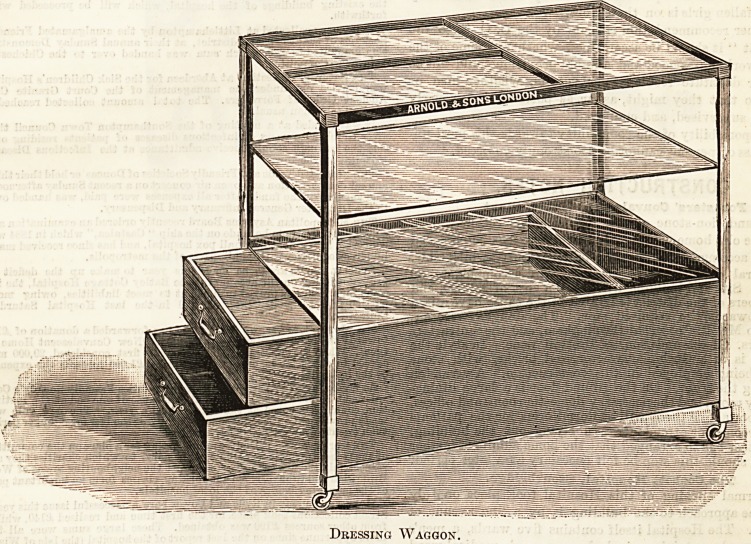# Dressing Waggon

**Published:** 1893-09-30

**Authors:** 


### PRACTICAL DEPARTMENTS.

### DRESSING WAGGON.

Our illustration shows a new idea for a dressing waggon, made by Messrs. Arnold and Sons, West Smithfield. The frame is of iron, enamelled white, and the three shelves are of plate glass. The two drawers, which are made to pull through, opening from either side, a very convenient arrangement, are lined throughout with copper. The top shelf is provided, as will be seen by the drawing, with divisions, which are of brass. The whole waggon is mounted on rubber castors, and can be moved with perfect ease. Messrs. Arnold's name is sufficient guarantee that this, like all their other surgical appliances, is thoroughly well finished in every particular, and deserves special commendation. The illustration is given by kind permission of the maker.

Dressing Waggon.

## Figures and Tables

**Figure f1:**